# A State Medical Service in Detail

**Published:** 1918-09-21

**Authors:** 


### The Hospital

The Workers' Newspaper of Administrative Medicine and Institutional Life, Administration, National Insurance and Health.

No. 1685, Vol. LXIV. SATURDAY, SEPTEMBER 21, 1918. PRICE TWOPENCE

### A STATE MEDICAL SERVICE IN DETAIL.

The term "a State Medical Service" has in recent controversies made many appearances, both in speech and print, and it has enjoyed the dignity and exercised the attraction which are the privilege of many sonorous and undefined phrases. Until a few years ago it was presented at frequent intervals by the "medical correspondent" of our leading daily newspaper, and, though the correspondent in question has now suffered eclipse, the banner is still held aloft by other hands. Doubtless for good and sufficient reasons the advocates of the proposal have generally abstained from translating their scheme into practical terms, and hence we have in these columns been little inclined to discuss the question. Until definitions are set down in black and white, argument, in these stern and strenuous days, is hardly worth while. It happens, however, that the past week has seen the circulation of proposals which are precise enough in all conscience, and which may well be taken as the outline of a State Medical Service in the sense in which the advocates of such an enterprise understand this term. The revelation proceeds from the Medico-Political Union, and the Council of this organisation are satisfied, from evidence in their possession, that the information which they announce is reliable. So far there is no open agitation on behalf of the scheme by its promoters, but it is alleged that general practitioners at present engaged on Army service are being canvassed in its favour. The suggestion is that these gentlemen, having become accustomed to an organised military Medical Service and to a definite annual income paid by the State, will favour the establishment of parallel conditions in civilian practice, and, further, that their adhesion to such a development will be quickened by the suspicion that on demobilisation they will return home only to find that their practices have largely disappeared. Here, it is supposed, is favourable ground for the organisation of medical support for a State Medical Service, and, given a certain measure of such support, it is doubtless anticipated that a start can be made and that gradually objectors will be forced to yield under the influence of the same kind of pressure as that which drove to surrender most of the medical opponents of the National Insurance Act. In a word, assuming the authenticity of the documents quoted—and such an assumption is not an extreme one—the report as printed and circulated may be accepted as what in detail certain reformers mean when they say that the salvation of the community and of the medical profession is to be secured by the establishment of a State Medical Service.

The principle of the scheme from a public point of view rests on the assumption that it is a proper function of the State to provide free medical benefits for all citizens who care to claim them. The intention, therefore, is to appoint in every locality such Government medical officers as may be judged necessary, to pay each of these officers an annual salary out of public funds, and to make the services of each available in the particular district where he is called upon to practise. Necessarily such an organisation involves various ranks and orders and various grades of pay. The classes suggested are five in number, and the payments proposed are to range from £400 to £1,500 per annum, the higher ranks of the Service being devoted mainly to administrative work or to consulting practice. There are other temptations for the practitioner. Drugs, appliances, clinical institutions, travelling expenses will not be to his charge, but will be paid for by the Government; study leave will be possible, and a month's holiday every year; evening surgery hours are to be abolished, and work after five o'clock will fall to the juniors; and at sixty years of age the practitioner will enjoy a pension equivalent to two-thirds of his salary. The hard-worked doctor may well sigh for times so smooth and for prospects so alluring. Indeed, there is something more, for he is told that the "big houses at present in vogue" will no longer be necessary. The social stamp will be superfluous for the doctor who boasts the Government *cachet*, and the side street and the small establishment will content the professional ambition. There is, however, a bitter element in this enticing draught. Between the neighbouring ranks of the hierarchy there stands a barrier in the shape of a professional examination, and he who covets promotion and something more than a modest £400 to £500 per annum must return to the juvenile trials of an earlier day. If he achieves success of this order, and is fortunate enough to attract the favour of his superior officers, he may hope to rise to such heights as the Service has to offer, and in due time, in his maturer years, to repose with dignity under the shadow of some wide-spreading beech tree.

What the medical profession will have to say to these proposals time will show. Obviously it is hoped that the money proposals will tempt the younger men. All we will say from this point of view is that any such scheme would entirely alter the existing atmosphere of professional life. The practitioner would lose much of his present freedom of thought and action, and would become part and parcel of an official machine, where, as is usual in such organisations, smoothness of working and absence of friction would be more valued than independence of judgment and initiative. But, whatever the medical profession may conclude, the last word on these proposals will be spoken by the British public. And, to us at all events, it will be a matter for surprise if the citizens of this country, even in these days of multiple regulations, will desire in times of sickness to replace their own selected medical adviser by a Government official who is guaranteed to be passing examinations at frequent intervals and who, when illness is so unreasonable as to occur after 5 P.M., will hand over the business to a junior member of the Service. Such a method of proceeding may be possible in the Army, where every patient is under military discipline and is trained to stand to attention when the medical officer enters the ward. But among free men and women another order prevails, and it is an order in which both doctor and patient may speak the thing he will. The scheme in detail admits of almost endless discussion, but, in our view, its central principle ignores the human relationships of medical practice, and we are convinced that the classes it would mainly concern will have none of it.

